# Comparative Analysis of Halitosis in Adolescents and Young Adults with Removable Retainers, Fixed Retainers, or No Orthodontic Treatment: A Cross-Sectional Study with Salivary pH Subgroup Analyses

**DOI:** 10.3390/jcm14103560

**Published:** 2025-05-19

**Authors:** Magda Mihaela Luca, Roxana Buzatu, Bogdan Andrei Bumbu

**Affiliations:** 1 Department of Pediatric Dentistry, Faculty of Dental Medicine, “Victor Babes” University of Medicine and Pharmacy Timisoara, Eftimie Murgu Square 2, 300041 Timisoara, Romania; luca.magda@umft.ro; 2 Department of Dental Aesthetics, Faculty of Dental Medicine, “Victor Babes” University of Medicine and Pharmacy Timisoara, Revolutiei Boulevard 9, 300041 Timisoara, Romania; 3 Department of Dental Medicine, Faculty of Medicine and Pharmacy, University of Oradea, 410073 Oradea, Romania; bogdanbumbu@uoradea.ro

**Keywords:** halitosis, orthodontic retainers, adolescent, saliva, quality of life

## Abstract

**Background and Objectives**: Halitosis is a persistent oral health issue that can undermine self-esteem and social interactions, particularly in younger populations who may be more vulnerable to peer judgment. Orthodontic retainers—both removable and fixed—can alter oral microbiota and salivary parameters, potentially influencing malodor development. This study aimed to compare halitosis severity and oral-health-related quality of life (OHRQoL) in adolescents and young adults (aged 12–25) wearing removable retainers, fixed retainers, or no orthodontic appliances, with an additional focus on salivary pH as a possible modifying factor. **Methods**: A total of 88 participants were allocated into three groups: removable retainer (n = 28), fixed retainer (n = 30), and no orthodontic treatment (n = 30). Halitosis severity was measured via organoleptic evaluation (0–5 scale) and the Halitosis Associated Life-Quality Test (HALT, 0–100). Salivary pH was determined using a digital pH meter. OHRQoL was assessed through the Oral Health Impact Profile–14 (OHIP-14, 0–56). One-way ANOVA with Tukey’s post hoc test and chi-square analyses were employed to compare outcomes among groups. Spearman’s correlation explored relationships among HALT, organoleptic scores, OHIP-14, and salivary pH. **Results**: Fixed retainer wearers exhibited higher mean organoleptic scores (2.2 ± 0.6) compared to removable retainer users (1.7 ± 0.5, *p* = 0.003). HALT results similarly showed that the fixed retainer group (35.6 ± 6.4) reported more halitosis-related burdens than the removable group (31.4 ± 5.9, *p* = 0.015). Low salivary pH (<6.8) was linked to greater malodor indices in all cohorts (*p* < 0.05). Correlations revealed moderate positive associations between HALT and OHIP-14 (r = +0.52, *p* < 0.001). **Conclusions**: Adolescents and young adults wearing fixed orthodontic retainers reported more severe halitosis and a correspondingly lower oral-health-related quality of life than those with removable retainers or no orthodontic appliances. Salivary pH emerged as an influential factor, indicating that maintaining a neutral oral environment could mitigate malodor. Targeted interventions emphasizing hygiene and saliva management may improve overall well-being in this vulnerable age group.

## 1. Introduction

Halitosis, commonly described as unpleasant breath odor, is a multifaceted condition with significant social and psychological consequences [1,2]. Global prevalence estimates suggest that between 22% and 50% of individuals may experience halitosis at varying degrees of severity [3,4]. Adolescents and young adults, in particular, can exhibit heightened sensitivity to peer perceptions, which may magnify the impact of malodor on self-esteem and interpersonal dynamics [5]. This heightened concern underscores the need for targeted research that explores both clinical and psychosocial dimensions of halitosis in younger populations.

Orthodontic treatment frequently involves the use of retainers after active tooth movement, which can be either removable or fixed (bonded to the lingual surfaces) [6]. While these appliances are essential for maintaining orthodontic results, they can create niches for bacterial growth and plaque accumulation, potentially elevating the risk of malodor [7,8]. Differences in retainer design and ease of cleaning—particularly between removable and fixed types—warrant a closer examination of their association with halitosis [9]. As the oral microbiome is highly sensitive to changes in local environment, additional colonization on retainer surfaces could contribute to odor production over time [10,11].

Saliva serves a crucial protective role within the oral cavity by providing both mechanical cleansing and antimicrobial actions [12]. Its pH also influences bacterial composition, with a more acidic environment fostering the proliferation of anaerobes known to produce volatile sulfur compounds (VSCs) [13,14]. Orthodontic appliances, including retainers, may alter salivary flow rate and pH, leading to shifts in the oral microbiota [15]. Thus, evaluating salivary pH can offer valuable insights into how these local ecological changes might affect malodor severity in individuals wearing removable or fixed retainers [16,17].

Adolescents and young adults, typically up to 25 years old, represent a demographic navigating unique social pressures and heightened self-awareness [18]. When orthodontic retention is introduced—whether through removable or fixed retainers—daily hygiene routines become more complex. Insufficient cleaning or lack of compliance in wearing removable retainers may further predispose this group to oral malodor [9]. Moreover, concerns about breath odor in these formative years can extend beyond clinical parameters, impacting overall oral-health-related quality of life (OHRQoL) [19]. Despite its significance, limited research systematically compares halitosis and OHRQoL outcomes between different orthodontic retention methods in this age group.

The psychosocial burden of halitosis can be measured using condition-specific instruments like the Halitosis Associated Life-Quality Test (HALT), which evaluates self-perceived social limitations, anxiety, and embarrassment arising from malodor [20]. Meanwhile, the Oral Health Impact Profile–14 (OHIP-14) provides a broader assessment of how oral conditions influence functional limitations, pain, and psychological discomfort. By integrating HALT and OHIP-14, researchers can gain a comprehensive view of how halitosis affects both general and halitosis-specific quality of life in younger populations [18,20].

The purpose of this cross-sectional study was threefold: (1) to quantify halitosis severity among adolescents and young adults wearing removable retainers, fixed retainers, or no orthodontic appliances; (2) to determine how salivary pH correlates with malodor; and (3) to assess the psychosocial impact of halitosis through HALT and OHIP-14 measurements. Identifying these relationships can inform tailored oral care strategies aimed at minimizing halitosis and fostering confidence in this formative age group [7,9].

## 2. Materials and Methods

### 2.1. Study Design and Ethics

This cross-sectional study was conducted at the “Victor Babeș” University of Medicine and Pharmacy in Timișoara between March 2023 and March 2025. Approval for the research was obtained from the Institutional Research Board, adhering to the principles outlined in the Declaration of Helsinki (approval code E-787, dated 8 February 2023). Prior to enrollment, all participants were informed about the study’s purpose and procedures, and written informed consent was collected. For individuals under the age of 18, consent was obtained from a parent or legal guardian, in accordance with national legislation (Article 167 of Law No. 95/2006 and Article 28, Chapter VIII of Order 904/2006).

The inclusion criteria were as follows: (1) adolescents and young adults aged 12 to 25 years; (2) individuals who wore a removable retainer or a fixed retainer for at least six months, or those who had no orthodontic appliance; (3) willingness to participate and provide informed consent or assent; (4) absence of any active orthodontic treatment, defined as being either in the retention phase or with no history of orthodontic therapy; and (5) availability to attend the clinics for scheduled examinations and data collection.

The exclusion criteria included the following: (1) antibiotic usage in the past 30 days; (2) active periodontal disease, defined as probing depths of ≥4 mm in at least three teeth and/or bleeding on probing in multiple quadrants; (3) significant systemic illnesses affecting saliva production, such as Sjögren’s syndrome or uncontrolled diabetes; (4) known allergies or sensitivities to typical dental materials; (5) use of medications known to reduce salivary flow, including antihistamines and tricyclic antidepressants; and (6) individuals currently wearing other orthodontic appliances, such as braces or functional appliances.

### 2.2. Participants and Group Allocation

All assessments were carried out in a dedicated, ventilated operatory 30 min after surface disinfection to avoid confounding ambient VSCs, with room conditions (22 ± 1 °C; RH ≈ 50%). Participants abstained from food, beverages (water allowed until 2 h beforehand), toothbrushing, and all mouthrinses for ≥24 h. Two examiners (M.M.L. and B.A.B.) independently rated organoleptic odor; every participant received two scores 15 min apart, and the mean was analyzed. Duplicate scoring on every third participant (n = 29) yielded inter-rater ICC = 0.84 (95% CI 0.72–0.91); intra-rater ICC (10 volunteers re-tested 1 week later) = 0.88.

A total of 88 eligible participants were enrolled and allocated into three groups based on their orthodontic status: removable retainer group (n = 28), fixed retainer group (n = 30), and no orthodontic treatment group (n = 30). The sample size of at least 80 participants was determined through a priori power analysis, indicating that 80 participants would be sufficient to detect a medium effect size (Cohen’s d = 0.5) with 80% power at a 5% significance level.

At recruitment, participants underwent a standardized interview to gather demographic data (age, sex) and oral hygiene habits (e.g., toothbrushing frequency, use of mouthrinses, type of toothpaste). Additional lifestyle information, including dietary habits and snacking frequency, was recorded to identify possible influences on salivary pH. A thorough intraoral examination confirmed the absence of active periodontal issues, evaluated for retainer wear status, and ensured compliance with the inclusion and exclusion criteria. Participants in the removable retainer group were asked to bring their retainer to every visit to confirm consistent usage and condition.

### 2.3. Halitosis Measurement and Salivary pH Assessment

Halitosis severity was quantified through a two-step process. (1) Organoleptic Evaluation: A single, calibrated examiner performed the organoleptic assessment using a 0–5 scale. The participants were instructed to keep their mouths closed for two minutes and then exhale slowly through a 10 cm tube. The examiner, blinded to group assignment, scored the odor intensity based on a standardized rubric. Inter- and intra-examiner reliability was assessed at the beginning of the study by having the examiner perform pilot measurements on 10 volunteers, achieving an intraclass correlation coefficient (ICC) > 0.80 for test–retest consistency. (2) Halitosis Associated Life-Quality Test (HALT): All participants completed the HALT questionnaire, consisting of 20 items scored on a 0–5 Likert scale (total score range of 0–100). Higher scores indicate greater psychosocial distress related to malodor. To maintain privacy and promote honest responses, each participant answered the questionnaire in a quiet area without the presence of clinic staff or peers.

Unstimulated saliva samples were collected to measure salivary pH. Participants were asked to refrain from food, beverages (other than water), and oral hygiene procedures (e.g., toothbrushing, mouthrinse use) for at least one day prior to testing. The 24 h interval was selected because (a) residual CHX/CPC substantivity falls sharply after 12–16 h and (b) longer deprivation was judged unethical in adolescents. They then expectorated unstimulated saliva into a sterile container for five minutes. A digital pH meter (accuracy ±0.1) was used to measure salivary pH immediately. The values were categorized as follows: (1) acidic: pH < 6.8; (2) near-neutral: pH 6.8–7.2; (3) slightly alkaline: pH > 7.2. This classification facilitated subgroup analyses to explore potential correlations between salivary pH and halitosis severity.

### 2.4. OHRQoL Assessment

Oral-health-related quality of life (OHRQoL) was assessed using the Oral Health Impact Profile–14 (OHIP-14). Each of the 14 items is scored on a 5-point Likert scale (0 = never, 4 = very often), yielding a total possible score of 0–56. Higher OHIP-14 scores suggest a greater negative impact of oral conditions on daily life. Participants were given the questionnaire in a quiet environment with instructions to answer candidly. Any clarifications on terminology were provided by trained research personnel.

### 2.5. Statistical Analysis

All collected data were entered into SPSS version 28 (IBM, Armonk, NY, USA). Descriptive statistics (means, standard deviations, frequencies) were computed for continuous and categorical variables. One-way analysis of variance (ANOVA) with Tukey’s post hoc tests assessed between-group differences in HALT, organoleptic scores, OHIP-14, and salivary pH for normally distributed variables. When distribution assumptions were not met, the Kruskal–Wallis test was used as a nonparametric alternative. Categorical variables (e.g., pH category, sex) were analyzed using the chi-square or Fisher’s exact test. Spearman’s correlation coefficients examined potential relationships among HALT scores, organoleptic measures, OHIP-14 scores, and salivary pH. All statistical tests were two-tailed, and significance was established at *p* < 0.05.

## 3. Results

Table 1 displays the demographic and basic oral hygiene data for the three groups: removable retainer users (n = 28), fixed retainer users (n = 30), and individuals with no orthodontic treatment (n = 30). Mean ages ranged between 17.4 and 18.0 years (*p* = 0.538), indicating a relatively homogenous distribution in this adolescent to young adult bracket. The proportion of females hovered around 57–60% across all cohorts (*p* = 0.948), minimizing potential gender-related biases in halitosis outcomes.

Brushing frequency averaged 1.8–2.0 times per day among the groups (*p* = 0.108), suggesting comparable oral hygiene habits. Similarly, mouthrinse usage was consistent—60.7% in the removable group, 60.0% in the fixed group, and 53.3% in the no treatment group (*p* = 0.747).

**Figure 1:**
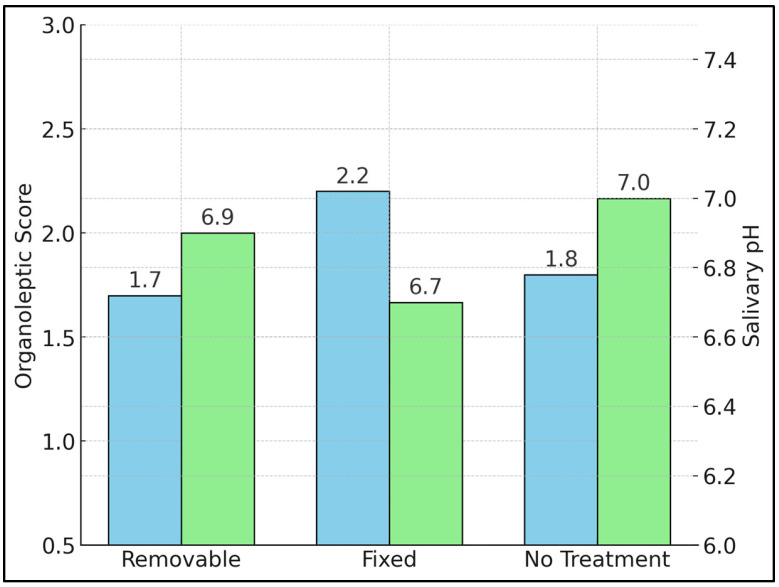
Organoleptic scores (blue color) and salivary pH (green color) in Removable, Fixed, and no Treatment Groups.

Table 2 compares the mean organoleptic scores (0–5) and salivary pH values among the removable retainer group, the fixed retainer group, and participants with no orthodontic treatment. The fixed retainer cohort registered the highest average organoleptic score of 2.2 ± 0.6, significantly exceeding both the removable group (1.7 ± 0.5, *p* = 0.003 post hoc) and the untreated group (1.8 ± 0.5, *p* = 0.014 post hoc). Salivary pH also differed significantly across the groups (*p* = 0.009). The fixed retainer group exhibited the most acidic saliva (mean pH 6.7 ± 0.3) compared to near-neutral levels in the removable group (6.9 ± 0.2) and the untreated controls (7.0 ± 0.3), as presented in Figure 1. Lower salivary pH is associated with an oral environment favorable to odor-producing anaerobes, possibly explaining the higher organoleptic readings in this group.

**Figure 2:**
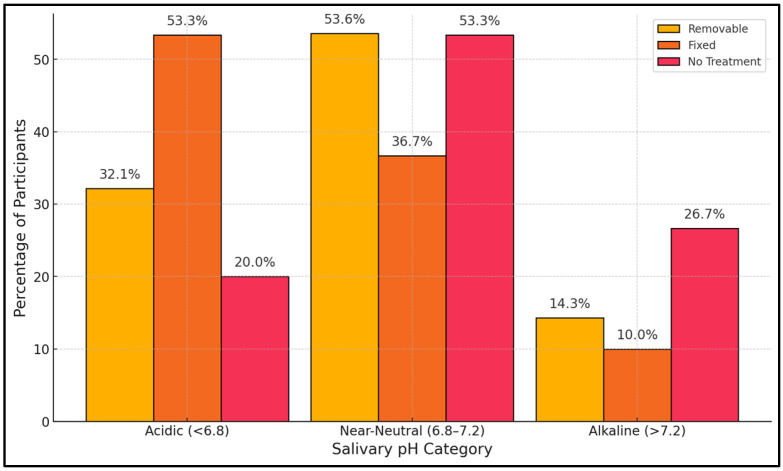
Salivary pH category.

Table 3 presents how participants are distributed according to salivary pH ranges: acidic (<6.8), near-neutral (6.8–7.2), and alkaline (>7.2). A chi-square test revealed a significant difference among the groups (*p* = 0.003). The fixed retainer group had the largest proportion of acidic saliva (53.3%), surpassing both the removable group (32.1%) and the untreated group (20.0%). Near-neutral pH (6.8–7.2) was the most frequent category overall, especially for removable retainer users (53.6%) and the no treatment group (53.3%). Interestingly, individuals in the no treatment cohort had the highest incidence of alkaline saliva (26.7%) compared to 14.3% for removable retainer wearers and only 10.0% for fixed retainer wearers (Figure 2).

Table 4 shows the mean Halitosis Associated Life-Quality Test (HALT) scores across four domains—Emotional Impact, Social/Interaction, Functional Discomfort, and Physical Concerns—alongside the total HALT. The fixed retainer group consistently displays the highest domain scores, particularly in Emotional Impact (11.4 ± 2.6) and Social/Interaction (10.1 ± 2.7). Although the removable group and the no treatment group posted lower scores overall, the differences in Emotional and Social dimensions between fixed and removable retainer groups reached statistical significance (*p* < 0.05). The Physical Concerns domain did not vary significantly among cohorts (*p* = 0.180), suggesting that the physical sensations related to halitosis (e.g., dry mouth, bad taste) may be comparatively uniform. However, the total HALT score was significantly higher in fixed retainer wearers (35.6 ± 6.4) than in removable retainer users (31.4 ± 5.9, *p* = 0.015 in post hoc) and slightly higher than in no treatment participants (33.5 ± 6.1, *p* = 0.067).

Table 5 provides an overview of the Oral Health Impact Profile–14 (OHIP-14) scores, examining specific domains (Functional Limitation, Psychological Discomfort, Physical Pain, Handicap) and the overall total. Generally, the fixed retainer group manifests slightly higher values across most domains, notably Functional Limitation (2.3 ± 1.0) and Psychological Discomfort (2.6 ± 1.2). Although the difference in Psychological Discomfort (*p* = 0.094) did not reach statistical significance, the Functional Limitation domain stands out (*p* = 0.041). The total OHIP-14 score was highest in the fixed retainer cohort (14.1 ± 3.5), contrasting with the removable group (12.5 ± 3.4) and the untreated group (12.3 ± 3.0).

Table 6 displays Spearman’s correlation coefficients linking four key metrics: HALT Total, organoleptic scores, salivary pH, and OHIP-14 Total. A moderate positive correlation exists between HALT and organoleptic values (r = +0.54, *p* < 0.001), suggesting that as clinically perceived malodor intensifies, adolescents and young adults report heightened psychosocial distress. HALT also correlates moderately with OHIP-14 (r = +0.52, *p* < 0.001).

**Figure 3:**
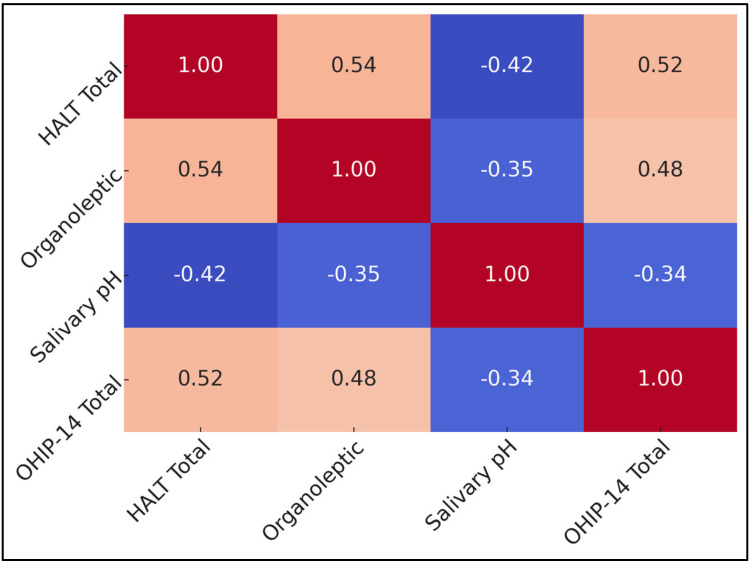
Correlation matrix heatmap.

Conversely, salivary pH demonstrates negative associations with both HALT (r = −0.42, *p* < 0.001) and organoleptic scores (r = −0.35, *p* < 0.001), indicating that more acidic conditions align with worse malodor, both in subjective and objective terms. There is a negative correlation between pH and OHIP-14 (r = −0.34, *p* < 0.001), as presented in Figure 3.

Table 7 contrasts participants with acidic saliva (pH < 6.8) against those whose saliva is near-neutral or alkaline (pH ≥ 6.8), focusing on mean HALT and organoleptic scores. Out of the 88 total participants, 31 presented with acidic saliva, reporting substantially higher HALT values (36.7 ± 6.3) and organoleptic scores (2.3 ± 0.6) compared to their counterparts (32.1 ± 5.8 and 1.8 ± 0.5, respectively). Independent t-tests revealed a statistically significant gap (*p* = 0.004), underscoring the influence of salivary acidity on both subjective and objective measures of malodor.

## 4. Discussion

### 4.1. Literature Findings

This cross-sectional study explored the interplay among orthodontic retainer type, salivary pH, and halitosis in adolescents and young adults. Our findings indicate that fixed retainers correlate with a higher prevalence of acidic saliva and more severe malodor, both clinically (organoleptic scores) and subjectively (HALT). In contrast, removable retainer wearers demonstrated somewhat milder levels of halitosis, possibly due to the device’s lower plaque-retentive potential and the ease of removing it for cleaning. Participants without orthodontic appliances tended to show near-neutral or slightly alkaline salivary pH, aligning with relatively lower halitosis indicators.

Salivary pH emerged as a critical modifier, with acidic conditions linked to elevated HALT and organoleptic scores across all groups. This relationship underscores how oral chemistry can shape bacterial ecology, enabling odor-causing microbes to thrive. Our correlation analysis showed that halitosis severity aligns with psychological distress, as measured by HALT and OHIP-14. These data affirm that beyond a mere biological phenomenon, malodor can substantially erode young individuals’ social confidence and emotional well-being, especially in an age group prone to heightened self-consciousness.

Taken together, the evidence suggests an integrated approach to halitosis management among adolescent and young adult retainer wearers. Oral health professionals should emphasize salivary pH assessment, regular retainer cleaning, and periodic professional follow-up to detect early signs of malodor. Removable retainers appear to pose fewer halitosis risks, but consistent hygiene remains essential. Interventions that aim to maintain or restore neutral pH, e.g., dietary modifications, saliva stimulants, and improved brushing protocols, might mitigate both the clinical and psychosocial burdens of malodor. Prospective, longitudinal research could further elucidate how retainer duration, evolving dentition, and ongoing maturity influence these outcomes over time.

In a similar manner to the study by Settineri et al. [21], which found significant associations between self-reported halitosis and various factors, such as relational dental anxiety, alcohol consumption, and poor oral hygiene, among others, in an Italian cohort (ORs ranging from 0.39 to 1.04), the research by Elif Yıldızer Keriş and colleagues also explored the impact of orthodontic appliances on halitosis [22]. However, unlike Settineri et al.’s findings that linked behavioral and psychological factors with halitosis, Keriş’s study, conducted on a younger demographic using fixed and removable space maintainers, concluded that these appliances did not significantly alter oral health status or halitosis over time, with all *p*-values exceeding 0.05 [21,22]. This suggests that while adult halitosis can be influenced by psychological and hygiene-related factors, in children, the type of orthodontic appliance might not have a measurable impact on halitosis, indicating a possible age-related difference in the factors contributing to halitosis. Both studies underline the complexity of halitosis, which is influenced by a variety of biological, psychological, and care-related factors that manifest differently across age groups.

In the study by Arwa A Banjar et al. [7], 41.5% of young adults undergoing orthodontic treatment reported experiencing halitosis, with a significant 62.5% noticing the issue during or after their treatment. Furthermore, a statistically significant correlation was identified, highlighting that regular dental visits were associated with a reduction in halitosis among those with fixed orthodontic appliances. In contrast, the systematic review by Raluca Briceag et al. [23] compiled results from various studies, revealing that the prevalence of self-reported halitosis among adolescents and young adults ranged significantly, from 23.1% to 77.5%, with an average of 44.7%. This review also noted psychological effects, as those affected reported increased feelings of anxiety and depression, measured using tools like the OHIP-14 and SCL-9-R. These findings emphasize the pervasive impact of halitosis not just on physical dental health but also on the emotional and social well-being of individuals, highlighting the necessity of comprehensive care strategies that include both dental treatment and psychological support to manage halitosis effectively.

In examining the interplay between orthodontic appliances and oral health, Lucchese et al. [24] conducted a systematic review that revealed that the use of orthodontic appliances significantly altered the oral microbiota, with a notable increase in cariogenic and periodontopathogenic bacteria, such as Streptococcus mutans, Lactobacillus spp., and various Gram-negative species, like Porphyromonas gingivalis and Fusobacterium nucleatum, suggesting a heightened risk for caries and periodontal disease during orthodontic treatment. In a similar manner, the longitudinal prospective study by Gámez Medina et al. [25] assessed the effects of fixed retainers on oral-health-related quality of life (OHRQoL) and found that upper retainers negatively impacted OHRQoL at baseline (*p* = 0.018), with overall OHIP-14 scores worsening over time (T0 to T1, *p* = 0.014). Notably, fractures or debonding of retainers were significantly associated with poorer patient-reported outcomes (*p* = 0.05). While Lucchese et al. focused on the microbiological implications of appliance use, Gámez Medina et al. provided patient-centered evidence indicating that mechanical complications may have long-term psychosocial consequences. Together, these studies underscore the importance of comprehensive clinical management during the retention phase, not only for microbial control but also to preserve patients’ quality of life.

Our findings accord with Kanzow et al. [26], who demonstrated that even transient pH reductions (<6.8) intensified VSC release after 4 h of mask wearing in young adults. Likewise, Muniz et al. [27] reported a 51% prevalence of self-reported malodor in institutionalized adolescents, driven by cariogenic burden and psychosocial stressors. These population data reinforce our correlation between HALT, OHIP-14, and organoleptic scores.

Short-term interventional work also suggests that restoring pH toward neutrality ameliorates odor. A randomized crossover trial using lemon essential oil mouthrinses raised salivary pH by ≈ 0.3 units and suppressed VSCs for 60 min [28]. The present study extends those physiological observations by linking chronic acidic profiles to quality of life decrements in retainer wearers.

Stress-related cortisol and heightened gingival inflammation reshape the plaque proteome, enriching *Fusobacterium*, *Prevotella,* and Gram-negative anaerobes that liberate hydrogen–sulphide and methyl–mercaptan. This ecological drift, documented in longitudinal adolescent cohorts with fixed appliances, offers a plausible biological bridge between our salivary pH signal and clinical malodor. Finally, our 24 h mouth-wash wash-out aligns with pharmacodynamic data showing that CHX/CPC substantivity falls below bactericidal thresholds within 12–16 h, making a one-week exclusion unnecessary while preserving ecological validity.

### 4.2. Study Limitations

This cross-sectional design precludes causal inference; longitudinal tracking of salivary pH and odor after retainer bonding is warranted. Second, although duplicate examiner scoring improved reliability, organoleptic testing retains subjectivity; portable gas-chromatography would strengthen future work. Third, residual CHX/CPC could not be fully excluded despite the 24 h wash-out, potentially under-estimating malodor in frequent rinsers. Fourth, the sample was derived from a single academic center and may not represent rural adolescents with differing fluoride access, diet, and socio-economic stressors.

## 5. Conclusions

Within this sample of 88 adolescents and young adults, fixed orthodontic retainers were associated with more pronounced halitosis, reflected in higher organoleptic scores and Halitosis Associated Life-Quality Test (HALT) ratings, than removable retainers or no orthodontic treatment. Salivary pH emerged as a pivotal factor; participants with acidic saliva (<6.8) consistently reported more severe malodor and greater psychosocial distress. Consequently, the findings underscore the critical role of oral environment management—in particular, maintaining a neutral to slightly alkaline pH—in mitigating the burdensome effects of halitosis among younger populations. Chair-side salivary pH assessment can serve as a research adjunct or a confirmatory tool when clinical malodor is suspected in retainer wearers, but we do not advocate for universal screening in routine orthodontic follow-up. From a clinical perspective, these results point to the importance of rigorous hygiene protocols for fixed retainers, including targeted brushing around bonded wires and professional monitoring. Removable retainers, while not entirely free from odor risk, appear less prone to harboring odor-causing bacteria, especially in the presence of healthy salivary composition.

## Figures and Tables

**Table 1:** Participant demographics.

Variable	Removable (n = 28)	Fixed (n = 30)	No Treatment (n = 30)	*p*-Value
Age (years, mean ± SD)	17.4 ± 2.2	18.0 ± 2.3	17.6 ± 2.4	0.538 (1)
Female, n (%)	16 (57.1)	18 (60.0)	17 (56.7)	0.948 (2)
Male, n (%)	12 (42.9)	12 (40.0)	13 (43.3)	–
Brushing frequency (times/day)	1.9 ± 0.4	1.8 ± 0.5	2.0 ± 0.4	0.108 (1)
Mouthrinse use (yes, %)	17 (60.7)	18 (60.0)	16 (53.3)	0.747 (2)
CHX 0.12%, n (%)	6 (21.4)	7 (23.3)	5 (16.7)	0.805 (2)
CPC 0.05%, n (%)	7 (25.0)	8 (26.7)	6 (20.0)	0.820 (2)
Essential oil blend, n (%)	4 (14.3)	3 (10.0)	5 (16.7)	0.748 (2)

(1) One-way ANOVA; (2) chi-square test; regular mouth-wash use (>3 d week) is common in Romania because national orthodontic recall leaflets recommend CHX or CPC rinses for plaque control in adolescents.

**Table 2:** Organoleptic scores and salivary pH.

Group	Organoleptic Score (Mean ± SD)	Salivary pH (Mean ± SD)
Removable (n = 28)	1.7 ± 0.5	6.9 ± 0.2
Fixed (n = 30)	2.2 ± 0.6	6.7 ± 0.3
No Treatment (n = 30)	1.8 ± 0.5	7.0 ± 0.3
*p*-value (ANOVA)	0.001	0.009

Post hoc (Tukey) for total HALT: aligners vs. lingual: *p* = 0.001; aligners vs. controls: *p* = 0.039; lingual vs. controls: *p* = 0.040.

**Table 3:** Distribution of salivary pH categories.

Salivary pH Category	Removable (n = 28)	Fixed (n = 30)	No Treatment (n = 30)	*p*-Value
Acidic (<6.8)	9 (32.1%)	16 (53.3%)	6 (20.0%)	0.003 (1)
Near-Neutral (6.8–7.2)	15 (53.6%)	11 (36.7%)	16 (53.3%)	–
Alkaline (>7.2)	4 (14.3%)	3 (10.0%)	8 (26.7%)	–

(1) Chi-square test for differences in distribution across pH categories.

**Table 4:** HALT (Halitosis Associated Life-Quality Test) scores by group.

HALT Domain	Removable (n = 28)	Fixed (n = 30)	No Treatment (n = 30)	*p*-Value (ANOVA)
Emotional Impact (0–25)	9.8 ± 2.4	11.4 ± 2.6	10.2 ± 2.5	0.02
Social/Interaction (0–25)	8.5 ± 2.3	10.1 ± 2.7	9.0 ± 2.1	0.01
Functional Discomfort (0–25)	7.8 ± 2.0	9.1 ± 2.4	8.3 ± 2.1	0.048
Physical Concerns (0–25)	5.3 ± 1.6	6.0 ± 1.8	5.6 ± 1.5	0.18
Total HALT (0–100)	31.4 ± 5.9	35.6 ± 6.4	33.5 ± 6.1	0.015

**Table 5:** OHIP-14 (Oral Health Impact Profile) scores.

OHIP-14 Domain	Removable (n = 28)	Fixed (n = 30)	No Treatment (n = 30)	*p*-Value (ANOVA)
Functional Limitation (0–8)	1.7 ± 0.9	2.3 ± 1.0	1.8 ± 0.8	0.041
Psychological Discomfort (0–8)	2.1 ± 1.1	2.6 ± 1.2	2.2 ± 1.0	0.094
Physical Pain (0–8)	1.8 ± 0.8	2.0 ± 0.9	1.7 ± 0.7	0.283
Handicap (0–8)	1.1 ± 0.6	1.3 ± 0.7	1.0 ± 0.5	0.166
Total OHIP-14 (0–56)	12.5 ± 3.4	14.1 ± 3.5	12.3 ± 3.0	0.038

**Table 6:** Correlation matrix: HALT, organoleptic, salivary pH, and OHIP-14.

Variables	HALT Total	Organoleptic	Salivary pH	OHIP-14 Total
HALT Total	1	+0.54	−0.42	+0.52
Organoleptic Score	+0.54	1	−0.35	+0.48
Salivary pH	−0.42	−0.35	1	−0.34
OHIP-14 Total	+0.52	+0.48	−0.34	1

**Table 7:** Acidic vs. near-neutral/alkaline salivary pH: HALT and organoleptic scores.

pH Category	n	HALT (Mean ± SD)	Organoleptic (Mean ± SD)	*p*-Value
Acidic (<6.8)	31	36.7 ± 6.3	2.3 ± 0.6	–
Near-Neutral/Alkaline (≥6.8)	57	32.1 ± 5.8	1.8 ± 0.5	0.004 (1)

(1) Independent t-tests for HALT and organoleptic between pH < 6.8 and pH ≥ 6.8.

## Data Availability

Data availability is subject to hospital approval.

## References

[1] Rosenberg M. (2002). The science of bad breath. Sci. Am..

[2] Bollen C.M.L., Beikler T. (2012). Halitosis: The multidisciplinary approach. Int. J. Oral Sci..

[3] Silva M.F., Leite F.R.M., Ferreira L.B., Pola N.M., Scannapieco F.A., Demarco F.F., Nascimento G.G. (2018). Estimated prevalence of halitosis: A systematic review and meta-regression analysis. Clin. Oral Investig..

[4] Scully C. (2014). Halitosis. BMJ Clin. Evid..

[5] Quirynen M., Dadamio J., Van den Velde S., De Smit M., Dekeyser C., Van Tornout M., Vandekerckhove B. (2009). Characteristics of 2000 patients who visited a halitosis consultation center. J. Clin. Periodontol..

[6] Zhao M., Yu C., Su C., Wang H., Wang X., Weir M.D., Xu H.H.K., Liu M., Bai Y., Zhang N. (2024). Dynamic effects of fixed orthodontic treatment on oral health and oral microbiota: A prospective study. BMC Oral Health.

[7] Banjar A.A., Hassan S.M., Alyafi R.A., Alawady S.A., Alghamdi M.H., Baik K.M. (2022). Self-perceived halitosis among young adults undergoing orthodontic treatment. Int. J. Dent. Hyg..

[8] Zhao M., Liu M., Chen W., Zhang H., Bai Y., Ren W. (2020). Salivary microbial changes during the first 6 months of orthodontic treatment. PeerJ.

[9] Lyros I., Tsolakis I.A., Maroulakos M.P., Fora E., Lykogeorgos T., Dalampira M., Tsolakis A.I. (2023). Orthodontic Retainers—A Critical Review. Children.

[10] Kumbargere Nagraj S., Eachempati P., Uma E., Singh V.P., Ismail N.M., Varghese E. (2019). Interventions for managing halitosis. Cochrane Database Syst. Rev..

[11] Abdulraheem S., Paulsson L., Petrén S., Sonesson M. (2019). Do fixed orthodontic appliances cause halitosis? A systematic review. BMC Oral Health.

[12] Aylıkcı B.U., Colak H. (2013). Halitosis: From diagnosis to management. J. Nat. Sci. Biol. Med..

[13] Abdullah M.A., Alasqah M., Sanaa M.S., Gufran K. (2020). The Relationship between Volatile Sulfur Compounds and the Severity of Chronic Periodontitis: A Cross-sectional Study. J. Pharm. Bioallied Sci..

[14] Tomazoni F., Vettore M.V., Baker S.R., Ardenghi T.M. (2019). Can a School-Based Intervention Improve the Oral Health-Related Quality of Life of Brazilian Children?. JDR Clin. Transl. Res..

[15] Anu V., Madan Kumar P.D., Shivakumar M. (2019). Salivary flow rate, pH and buffering capacity in patients undergoing fixed orthodontic treatment—A prospective study. Indian J. Dent. Res..

[16] Bollen C.M.L., Quirynen M. (1996). Microbiological response to mechanical treatment in combination with adjunctive therapy. J. Clin. Periodontol..

[17] Jakavičė R., Žarovienė A. (2023). Changes in the pH and the Flow Rate of Saliva During Orthodontic Treatment with Fixed Orthodontic Appliances: A Systematic Review. Turk. J. Orthod..

[18] Briceag R., Caraiane A., Raftu G., Bratu M.L., Buzatu R., Dehelean L., Bondrescu M., Bratosin F., Bumbu B.A. (2023). Validation of the Romanian Version of the Halitosis Associated Life-Quality Test (HALT) in a Cross-Sectional Study among Young Adults. Healthcare.

[19] Slusanschi O., Moraru R., Garneata L., Mircescu G., Cuculescu M., Preoteasa E. (2013). Validation of a Romanian version of the short form of the oral health impact profile (OHIP-14) for use in an urban adult population. Oral Health Prev. Dent..

[20] Blom T., Slot D.E., Quirynen M., Van der Weijden G.A. (2012). The effect of mouthrinses on oral malodor: A systematic review. Int. J. Dent. Hyg..

[21] Settineri S., Mento C., Gugliotta S.C., Saitta A., Terranova A., Trimarchi G., Mallamace D. (2010). Self-reported halitosis and emotional state: Impact on oral conditions and treatments. Health Qual. Life Outcomes.

[22] Yıldızer Keriş E., Atabek D., Güngör K. (2016). Effects of fixed and removable space maintainers on halitosis. BMC Oral Health.

[23] Briceag R., Caraiane A., Raftu G., Horhat R.M., Bogdan I., Fericean R.M., Shaaban L., Popa M., Bumbu B.A., Bratu M.L. (2023). Emotional and Social Impact of Halitosis on Adolescents and Young Adults: A Systematic Review. Medicina.

[24] Lucchese A., Bondemark L., Marcolina M., Manuelli M. (2018). Changes in oral microbiota due to orthodontic appliances: A systematic review. J. Oral Microbiol..

[25] Medina M.C.G., Santos C.C.O.D., Lima B.O., Ferreira M.B., Normando D. (2024). Impact of fixed orthodontic retainers on oral health-related quality of life: A longitudinal prospective study. Dent. Press J. Orthod..

[26] Kanzow P., Rammert L.S., Rohland B., Barke S., Placzek M., Wiegand A. (2023). Effect of face masks on salivary parameters and halitosis: Randomized controlled crossover trial. J. Oral Pathol. Med..

[27] Muniz F.W.M.G., Moreno L.B., da Silviera T.M., Rösing C.K., Colussi P.R.G. (2023). Prevalence and associated factors of self-reported halitosis among institutionalized adolescents: Cross-sectional study. Int. J. Dent. Hyg..

[28] Ma L., Pang C., Yan C., Chen J., Wang X., Hui J., Zhou L., Zhang X. (2023). Effect of lemon essential oil on halitosis. Oral Dis..

